# Supplementary material for: Preoperative Serum Markers and Risk Classification in Intrahepatic Cholangiocarcinoma: A Multicenter Retrospective Study
Source: Cancers (Basel). 2022 Nov 7;14(21):5459. doi: 10.3390/cancers14215459 (PMC9658667; doi:10.3390/cancers14215459)
Supplement: Supplementary file 1 [file cancers-14-05459-s001.zip › cancers-1936524-supplementary.pdf]

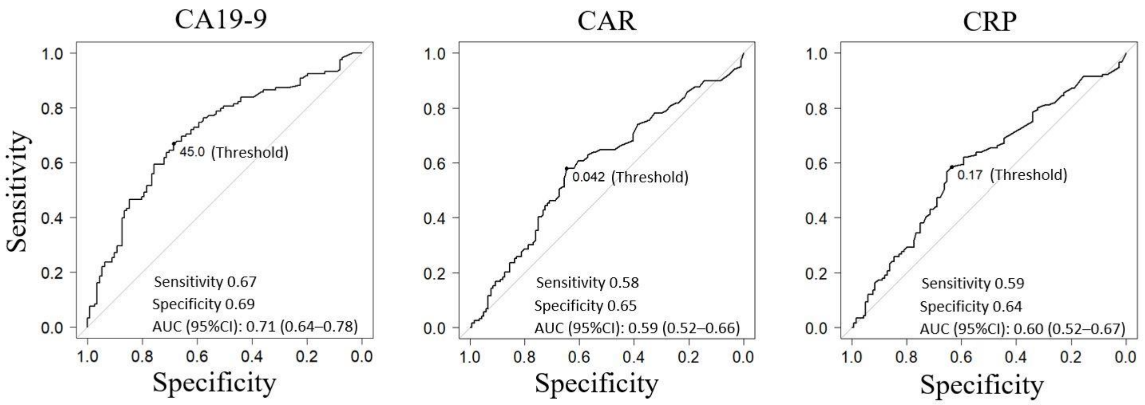

Supplementary Figure S1. ROC analysis of the value of preoperative prognostic factors with overall mortality as the outcome.
